# Acceptance of COVID-19 Vaccination in Cancer Patients in Hong Kong: Approaches to Improve the Vaccination Rate

**DOI:** 10.3390/vaccines9070792

**Published:** 2021-07-16

**Authors:** Wing-Lok Chan, Yuen-Hung Tricia Ho, Carlos King-Ho Wong, Horace Cheuk-Wai Choi, Ka-On Lam, Kwok-Keung Yuen, Dora Kwong, Ivan Hung

**Affiliations:** 1 Department of Clinical Oncology, LKS Faculty of Medicine, The University of Hong Kong, Hong Kong, China; triciaho@hku.hk (Y.-H.T.H.); lamkaon@hku.hk (K.-O.L.); dlwkwong@hku.hk (D.K.); 2 Department of Pharmacology and Pharmacy, LKS Faculty of Medicine, The University of Hong Kong, Hong Kong, China; carlosho@hku.hk; 3 Department of Family Medicine and Primary Care, LKS Faculty of Medicine, The University of Hong Kong, Hong Kong, China; 4 Department of Clinical Oncology, Queen Mary Hospital, Hong Kong, China; hcchoi@hku.hk (H.C.-W.C.); yuenkk1@hku.hk (K.-K.Y.); 5 Department of Medicine, LKS Faculty of Medicine, The University of Hong Kong, Hong Kong, China; ivanhung@hku.hk

**Keywords:** cancer, vaccination, acceptance, anxiety, COVID-19

## Abstract

Emerging efficacy and safety data have led to the authorization of COVID-19 vaccines worldwide, but most trials excluded patients with active malignancies. This study evaluates the intended acceptance of COVID-19 vaccination in cancer patients in Hong Kong. Methods: 660 adult cancer patients received a survey, in paper or electronic format, between 31 January 2021 and 15 February 2021. The survey included patient’s clinical characteristics, perceptions of COVID-19 and vaccination, vaccine knowledge, cancer health literacy, and Hospital Anxiety and Depression scale (HADS). The primary outcome was the intended acceptance of COVID-19 vaccine in cancer patients. Multivariable analysis was performed to identify factors associated with intended acceptance. Results: The intended acceptance of COVID-19 vaccination was 17.9%. A total of 487 (73.8%) believed that vaccination could prevent them from infection. Over 70% worried about vaccine negative effects on cancer and its side effects. Factors associated with intended acceptance included higher level of “belief in vaccine on preventing them from getting COVID-19”, less worry about long-term side effects of vaccine, lower level of cancer health literacy, and normal HADS (Depression scale). Conclusions: To improve vaccine acceptance rate, public education campaigns specific to cancer patients to gain their trust in efficacy and relieve their worries are needed.

## 1. Introduction

The coronavirus disease 2019 (COVID-19) has spread worldwide and become a pandemic of international concern since its emergence in December 2019. By 9 May 2021, there were over 158 million cumulative coronavirus cases and more than three million deaths [1]. COVID-19 has caused an unprecedented global burden on human health and the economy, as well as a tremendous impact on the psychosocial well-being of the population.

No effective treatment exists for COVID-19. Preventive measures including mask wearing, hand washing and social distancing are the only ways to prevent the spread of the virus. Universal vaccination is considered as the key successful measure against COVID-19. More than 60 vaccines for COVID-19 are either undergoing clinical trials or already approved for use in some countries. For example, BNT162b2 (Pfizer-BioNTech, Marburg, Germany) and mRNA-1273 (Moderna, U.S.), two messenger RNA vaccines, were approved by the US Food and Drug Administration for emergency use in the US; the ChAdOx1 nCoV-19 vaccine (Astra Zeneca Oxford, United Kingdom) was approved by the UK Medicines and Healthcare Products Regulatory Agency; Sinovac CoronaVac (company, city, country), an inactivated SARS-CoV-2 vaccine, was developed and authorized for use in China.

Hong Kong, which has a population of 7.5 million people, had recorded around 10,800 cases of COVID-19 in mid-February 2021, which was the time of the fourth wave. Two types of vaccine were also available in Hong Kong since mid-February 2021, namely, Sinovac CoronaVac (Inactivated whole virion vaccine) and Pfizer-BioNTech BNT162b2 (mRNA vaccine). Both vaccines are given free of charge to the population and people can choose either vaccine. At that time, Sinovac CoronaVac had not been approved by the World Health Organization (WHO) for emergency use.

Vaccination effectiveness to achieve herd immunity depends on the proportion of uptake by the population. After launching the vaccination programme, the Hong Kong Government has implemented multiple measures to improve the vaccination rate, including various rewarding programmes, granting vaccination leave, conducting outreach vaccination service, and facilitating gatherings after vaccination. Public education on the importance of vaccination is also provided on different media platforms including television, radio and newspapers. Despite these attractive measures and public education, the vaccination rate in Hong Kong remains low. By mid-May 2021, i.e., three months after the start of the COVID-19 vaccination programme in Hong Kong, only about 16.3% of the population had received at least one vaccine dose [2]. Many people are uncertain about vaccination. A study showed that the overall COVID-19 vaccine acceptance rate for Hong Kong adults was low (only 37%), and the key obstacle to acceptance was the lack of confidence in vaccine manufacturers [3].

According to the vaccination schedule proposed by the Hong Kong government, priority is given to people aged 60 years or above and those with chronic medical problems aged between 16 and 59 years [4]. Cancer patients are also included in the priority list and they can choose both Sinovac and BioNTech vaccines. Previous studies have shown that cancer patients have a higher risk of contracting COVID-19 and more severe complications with a higher mortality rate than the general population. They are more susceptible to infection because of a weakened immune system due to the cancer itself or the cancer treatment. A meta-analysis of 46,499 patients with COVID-19 showed that all-cause mortality was higher in patients with cancer (2034 deaths, risk ratio 1.66; 95% CI 1.33–2.07, *p* < 0.001), and the need for intensive care unit admission was also more likely in patients with cancer (3220 events, risk ratio 1.56; 95% CI 1.56; 95% CI, 1.33–2.07, *p* < 0.0001) [5].

Even though most efficacy data came from normal populations, a previous study showed that patients with cancer could develop high immunogenicity after two doses of vaccine [6]. Cancer patients, especially Chinese, often worry about adding extra medications on top of their cancer treatment. They worry if a new medication would affect their general well-being or cause adverse events on their cancer treatment. In this study, we aim to evaluate the intended acceptance of COVID-19 vaccination in cancer patients in Hong Kong and identify their concerns and the potential barriers to COVID-19 vaccination.

## 2. Materials and Methods

### 2.1. Study Setting and Design

This is a cross-sectional study conducted through an electronic or paper format. The survey was conducted between 31 January 2021 and 15 February 2021, which was the period just before the start of the COVID-19 vaccine implementation in Hong Kong. At the time of the survey, it was the fourth wave of COVID-19 in Hong Kong, and the number of confirmed cases was progressively increasing. The study participants were all adult cancer patients aged ≥18 years, who were either on cancer treatment, palliative care or cancer survivors. They gave consent to participant in this research study. There were two methods of patient recruitment: (1) recruitment from out-patient oncology clinics of the Department of Clinical Oncology in Queen Mary Hospital and Gleneagles Hospital, and (2) recruitment through different big cancer patient groups, including Cancer Information Network, Follow Grace, Hong Kong Breast Cancer Dragon Boat, Global Chinese Breast Cancer Organizations Alliance, etc. A link to the electronic survey was distributed via various methods, including a closed email system and secure social media platforms. With a cancer population of 15 million in Hong Kong, taking a 95% confidence interval and a 5% margin of error and assuming a 30% intended acceptance rate and a 75% response rate, we needed a minimum of 508 participants. The study protocol was approved by the Institutional Review Board of the University of Hong Kong/Hospital Authority Hong Kong West Cluster (HKU/HA HKW IRB). A statement of informed consent was provided on the first page of the questionnaire (both paper and electronic formats), and all responses were anonymous.

### 2.2. Survey Questionnaire

The questionnaire was developed with a panel of clinical oncologists and statisticians. The first draft was given to five non-health care workers and 10 cancer patients to check and comment. Comments from these 15 individuals were then used to modify and finalize the questionnaire.

The questionnaire included several parts: (1) participant’s characteristics including gender, age, educational level, employment status, medical insurance, history of influenza vaccination, cancer type, stage of cancer, current cancer treatment; (2) anxiety about getting COVID-19 and cancer; (3) concern about COVID-19 vaccination, which included questions on the side effects of vaccines and their impact on cancer treatment; (4) a short quiz on the basic knowledge about COVID-19 vaccines (this part involved five basic questions to test participants’ knowledge about COVID-19 vaccines with answers easily available on social media); (5) cancer health literacy (“Cancer Health Literacy Test-6” (CHLT-6), which is a validated six-item tool, was used) [7]; (6) the Hospital Anxiety and Depression Scale (HADS), which is a validated tool commonly used to assess for the baseline depressive and anxiety level (a score ≥11 suggests further investigation for mood disorder) [8]; and (7) intended acceptance of and preference for a particular COVID-19 vaccine. The questionnaire was in Appendix A. For parts (2) and (3), four choices (strongly agree, agree, disagree and strongly disagree) were given for participants to choose.

### 2.3. Data Processing and Analyses

The primary study outcome was the intended acceptance of a COVID-19 vaccine, where patients responding as being “very likely” or “likely” to get a COVID-19 vaccine was counted as intended acceptance.

Regarding their attitudes and beliefs about COVID-19 infection and vaccination, the participants’ responses were grouped into two main categories: “certainly disagree” and “disagree” were in the “disagree” category, whereas “certainly agree” and “agree” were in the “agree” category. 

Categorical variables were summarized as frequencies and proportions, whereas continuous variables were summarized as mean and standard deviation (SD). Univariate and multivariable logistic regression was performed to evaluate the independent predictors of the intended acceptance of COVID-19 vaccine of cancer patients to receive a COVID-19 vaccine. For univariate analysis, t-test was used for comparing the means of the two groups and chi-square test for categorical variables. The predictors included in the multivariable regression model were gender, age (18–60 and >61), insurance coverage, stage of disease (stage I–III and IV), current cancer systemic treatment, perceived risk of getting COVID-19 infection, anxiety level on COVID-19 vaccine and its side effects, cancer health literacy and HADS score. Associations between knowledge level on COVID-19 vaccine in addition to aforementioned predictors and preference on vaccination types (preference versus no preference) were also evaluated using multivariable logistic regression. Missing data were excluded from the analysis.

Two-sided *p*-values less than 0.05 were considered statistically significant. All statistical analyses were performed using IBM SPSS Statistics for Windows, Version 25 (IBM, Armonk, NY, USA).

## 3. Results

Overall, 660 cancer patients participated in the survey: 302 (45.8%) were aged 60 years or above, 432 (65.5%) were female, 412 (62.4%) had an educational level below tertiary level, 283 (42.9%) had private insurance, 384 (58.2%) had stage IV cancer, 456 (69.1%) were currently on anticancer systemic treatment and 141 (21.4%) received flu vaccination in the past 12 months. The baseline characteristics are presented in Appendix B.

### 3.1. Acceptance of COVID-19 Vaccination by Cancer Patients

Overall, 118 participants (17.9%) indicated intended acceptance of COVID-19 vaccination, while 542 (82.1%) were either reluctant for vaccination (n = 297, 45%) or could not decide (n = 25, 37.1%). 

**Figure 1:**
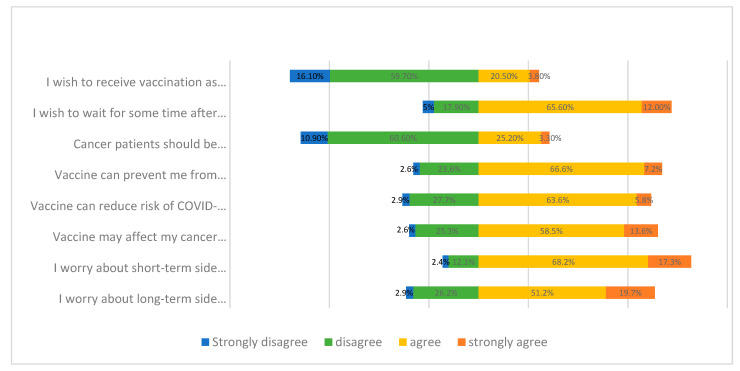
Perception of cancer patients on COVID-19 vaccination.

One hundred and fifty-five participants (24.3%) wished to receive the vaccine as soon as it was available, 188 (28.5%) considered that cancer patients should have the priority to be vaccinated and 512 (77.6%) would like to wait for some time after vaccine implementation. Responses of perception of COVID-19 vaccination are shown in Figure 1.

In univariable analysis, a significantly higher proportion of participants who were male, did not have private medical insurance, had a lower level of cancer health literacy and had normal HADS depression score (<11) were more willing to get vaccinated than the participants who were female, had medical insurance, higher level of cancer health literacy and had high HADS depression score (Table 1).

### 3.2. Perception of COVID-19 Pandemic

**Figure 2:**
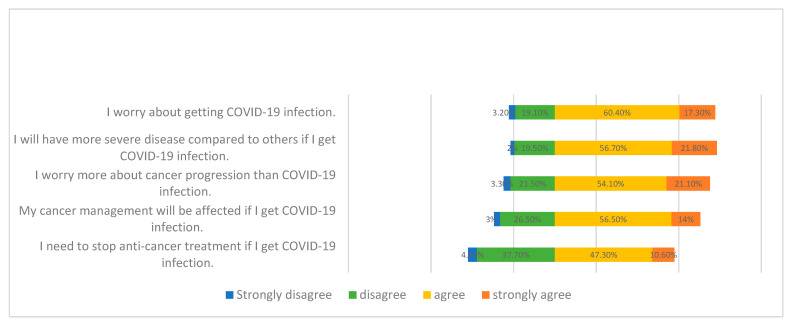
Responses of participants’ perception and concerns regarding COVID-19 pandemics.

The frequencies of responses revealing the participants’ perception and concerns regarding the COVID-19 pandemic are shown in Figure 2. 

Five hundred and thirteen participants (77.7%) expressed worry about being infected by COVID-19, whereas 518 participants (78.5%) believed that the severity of their COVID-19 infection could possibly be worse than those of other people.

Analysis of the participants’ concern regarding cancer progression and COVID-19 infection showed that 357 participants (54.1%) agreed and 139 (21.1%) strongly agreed that they worried more about cancer progression than COVID-19 infection, and 465 participants (70.5%) perceived a negative impact of COVID-19 on their cancer management, whereas 382 (58.9%) believed that their cancer treatment might need to be stopped.

Multivariable analysis was performed on “worry about getting COVID-19 infection” and “worry about cancer progression with COVID-19 infection”. There were no significant associations between these two perceptions with the intended acceptance of vaccination (Table 2).

### 3.3. Concerns about Cancer and COVID-19 Vaccination

Four hundred and eighty-seven participants (73.8%) trusted that a vaccine could prevent them from getting infected, and 458 (69.4%) perceived a lower chance of COVID-19 complications if vaccinated (Figure 1).

On the other hand, 476 participants (72.1%) were concerned about the negative effects of the vaccine on their cancer treatment, 564 (85.5%) worried about the short-term side effects of the vaccine and 468 (70.9%) worried about the long-term side effects (Figure 1).

### 3.4. Basic Knowledge on COVID-19 Vaccination

**Figure 3:**
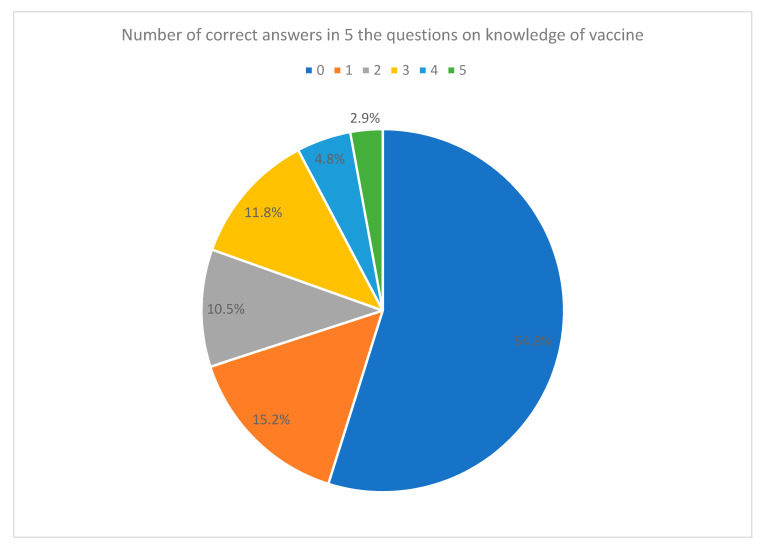
Pie chart on score distribution of knowledge about vaccine (number of correct answers).

Five questions were used to assess the participants’ knowledge on COVID-19 vaccination. The answers to these five questions were frequently reported on social media. Only 129 participants (19.5%) could get three or more correct answers, and 297 participants (45%) chose “unknown” for all five questions (Figure 3).

### 3.5. Independent Variables Associated with COVID-19 Vaccine Acceptance

Multivariate analysis was performed to assess the variables associated with COVID-19 vaccine acceptance in cancer patients, and the results are shown in Table 2. Participants who believed that the vaccine could prevent them from getting COVID-19 infection (odds ratio (OR) 2.97, 95% CI 1.59–5.56, *p* = 0.001) were more likely to accept COVID-19 vaccination, whereas those who had more worries about long-term side effects of vaccine (OR 0.46, 95% CI 0.28–0.76, *p* = 0.002), better cancer health literacy (OR 0.75, 95% CI 0.64–0.89, *p* = 0.001) and high HADS depression score (OR 0.49, 95% CI 0.29–0.82, *p* = 0.007) were less likely to accept vaccination. Factors including having private medical insurance and gender, which were significant in univariable analysis, were not significant in multivariable analysis.

### 3.6. Information Cancer Patients Want to Know

Concerning the information that participants would like to know before vaccination, the top was “effects of vaccine towards cancer treatment” (*n* = 539, 81.7%), followed by “side effects of the vaccine” (*n* = 383, 58.0%) and “efficacy of the vaccine” (*n* = 351, 53.2%). 

Comparing with the vaccine acceptance group, participants in the non-acceptance group would like to learn more about the “effects of vaccine towards cancer treatment” (*n*= 451, 82.3% vs. *n* = 88, 74.6%, *p* = 0.028). There was no significant difference on “efficacy of the vaccine” (*n*= 294, 54.2% vs. *n* = 57, 48.3%, *p* = 0.592) and “side effects of the vaccine” (*n*= 325, 60.0% vs. *n* = 58, 49.2%, *p* = 0.068). 

### 3.7. Vaccine Preference

Three hundred and seventy-six participants (57.0%) expressed having preference for a particular type of COVID-19 vaccine. In multivariate analysis, participants who were male (OR 0.68, 95% CI 0.47–0.97, *p* = 0.038), currently not on anticancer treatment (OR 0.66, 95% CI 0.45–0.98, *p* = 0.038), had better cancer health literacy level (OR 1.23, 95% CI 1.07–1.41, *p* = 0.004), better knowledge about vaccine (OR 1.38, 95% CI 1.21–1.57, *p* = 0.001) and normal HADS depression score (OR 0.64, 95% CI 0.44–0.92, *p* = 0.015) were more likely to have preference for a particular type of vaccine. The result is shown in Table 3.

## 4. Discussion

Our multicenter study describes the perceptions of cancer patients on getting COVID-19 vaccination. We documented an overall low intended acceptance for vaccination among cancer patients. Only 17.8% of the respondents were willing to receive a COVID-19 vaccine. Over 80% of the respondents had not decided yet or showed reluctance towards COVID-19 vaccination.

At the time of writing, we conducted a short cross-sectional study on the percentage of cancer patients who received a COVID-19 vaccine. We checked the medical records of all cancer patients who had oncology follow-up in our department from 3 May 2021 to 7 May 2021 (i.e., around three months after starting the vaccine programme in Hong Kong). Only 26 out of 628 patients (4.1%) had received at least one dose of COVID-19 vaccine. 

Our important findings include the following: (1) Most cancer patients worried about COVID-19 infection and its complications. (2) Multivariable analysis showed that variables significantly associated with intended acceptance of COVID-19 vaccinated included the belief that vaccination would prevent them from getting infection, less worry about long-term side effects of vaccine, lower cancer health literacy and normal HADS depression score. (3) Cancer patients, in general, were more concerned about their cancer management than COVID-19 infection. (4) Cancer patients expressed worry about the negative effect of the vaccine on their cancer treatment, as well as the short- and long-term side effects of the vaccine. (5) Over 80% of the cancer patients had limited knowledge about the COVID-19 vaccine. (6) The top information cancer patients wanted to know was “effects of vaccine towards cancer treatment”.

These findings reflected the reasons for the low acceptance of COVID-19 vaccination in cancer patients. To improve the acceptance rate and vaccination rate, and more importantly, to protect our cancer patients who are vulnerable to infection, we have the following humble suggestions.

### 4.1. Cancer Patients Should Be Well-Informed about the Risk of COVID-19

Cancer patients should be informed that they are at a higher risk of COVID-19 infection and health consequences owing to their weakened immune system caused by both tumor growth and cancer treatment.

In a study of 73 million patients in the USA, including 273,000 patients with underlying cancer, cancer patients had greatly increased odds of COVID-19 infection (adjusted OR of 7) [9]. Patients with cancer and COVID-19 had significantly worse outcomes (hospitalization, 47.46%; death, 14.93%) than patients with COVID-19 without cancer (hospitalization, 24.26%; death, 5.26%) (*p* < 0.001). In another study from Wuhan, China, which included 1524 patients with cancer at a single tertiary care hospital, cancer patients had a twofold increased risk of COVID-19 infection [10]. A study from Spain also showed that the cumulative incidence of COVID-19 was higher in cancer patients than in the general population (4.2% vs. 0.63%, *p* < 0.00001) [11].

Besides higher incidence, cancer patients are often older (i.e., aged ≥65 years), have one or more major comorbidities, poor functional status and more frequent hospital visits and admissions, putting them a higher risk of COVID-19-related morbidity and mortality.

In Liang et al.’s study, which analyzed 2007 cases from 575 hospitals in 31 provincial administrative regions in China, 39% of cancer patients with COVID-19 developed severe symptoms, compared with only 8% of non-cancer COVID-19 patients [12]. Patients who had chemotherapy or surgery one month before diagnosis of COVID-19 infection had a significantly higher risk of clinically severe events than those who did not receive cancer treatment (75% vs. 43%). Data from the COVID-19 and Cancer Consortium (CCC19) cohort study, which included 1018 cancer patients in US, also showed significantly higher risks of mortality and severe illness in COVID-19 cancer patients than in the general population [13]. Patients with advanced age, male sex, former smoking, presence of comorbidities, receiving chemotherapy using azithromycin plus hydroxychloroquine and poor Eastern Cooperative Oncology Group (ECOG) performance status and active (measurable) cancer were associated with increased 30-day all-cause mortality. Cancer patients should know about the higher risk of infection and understand the need for extra protection.

### 4.2. Cancer Patients Should Not Compare COVID-19 Infection with Cancer

A large proportion of patients had a misconception and compared the severities of COVID-19 infection and cancer. Indeed, infection is an acute condition, whereas cancer is a chronic disease.

COVID-19 should be viewed similarly to other types of acute infection. Cancer management can be likened to a marathon run. Patients need to have endurance and be persistent to achieve gradual progress. Any type of acute infection, be it COVID-19 or any other infection, can affect this long cancer battle. We, oncologists, should explain to patients and their care providers not to compare COVID-19 and cancer. Instead, we should emphasize that they are more vulnerable to infection and the infection may adversely affect their general condition as well as cancer treatment. Patients need to take extra precautions and exercise vigilance as cancer treatment may be stopped if infected.

Moreover, there is emerging evidence showing patients after COVID-19 infection continued to have ongoing symptoms many weeks and months after acute illness, often called “long-COVID” [14]. Patients with COVID-19 infection may have multi-organ damage that can persist months after discharge and the two common symptoms of long-COVID are fatigue and breathlessness [15,16]. A recent UK study also demonstrated gas transfer limitation in the lungs shown on hyperpolarized 129Xe MRI (XeMRI) imaging in nine patients with post-COVID-19 pneumonia [17]. Even though there is no solid evidence that cancer patients might have a high risk of these long-term sequelae, long-COVID will certainly impair cancer patients’ quality of life.

### 4.3. Despite the Limited Efficacy and Safety Data of COVID-19 Vaccines in Cancer Patients, International Health Organisations Already Recommend COVID-19 Vaccines to Cancer Patients Given the Well-Known Knowledge on Other Common Vaccines

Data on the safety, tolerability and efficacy of COVID-19 vaccines in cancer patients are limited owing to the exclusion of patients with active malignancies from most of the vaccination trials. For example, a study on the Sinovac CoronaVac excluded all people with a history of malignancy. In the trial of the BNT162b2 (Pfizer-BioNTech) vaccine, only 3.7% of the 43,549 participants enrolled were reported to have cancer [18].

Despite limited data, international medical societies and cancer networks, including the American Association for Cancer Research (AACR), American Society of Clinical Oncology (ASCO), European Society of Medical Oncology (ESMO), National Comprehensive Cancer Network (NCCN), Center for Disease Control and Prevention (CDC) and Spanish Medical Oncology Society (SEOM), have released positional statements and guidelines supporting vaccination in all cancer patients including those receiving active therapy in view of the greater severity and higher risk of mortality in cancer patients [19,20,21,22]. Some guidelines also set cancer patients as a high-priority subgroup for COVID-19 vaccination.

Good news on vaccine efficacy in cancer patients, from a UK study called “SOAP”, was recently released [23]. The anti-SARS-CoV-2 antibody responses improved from 39% after the first dose to 95% after the second dose given three weeks apart in cancer patients. By contrast, if the second dose was given after five weeks, only 43% of solid cancer patients and 8% of hematological cancer patients developed antibodies compared with 100% of the healthy controls. This study demonstrated that the COVID-19 vaccine is effective in cancer patients if the two doses are given in a timely manner.

Cancer patients on chemotherapy, targeted agents or immunotherapies often have a weakened immune system, which might make the vaccine less effective. The timing of the vaccination is important as this may affect its efficacy. On the basis of extrapolation from the recommendations concerning common vaccines such as the influenza vaccine, it is recommended that for patients who are on chemotherapy, a vaccine should be given 1–2 weeks before or 1–2 weeks after chemotherapy to increase the potential for the immune system to mount a response.

In terms of safety, COVID-19 vaccines undoubtedly have common short-term side effects including pain, redness or swelling at the injection site, malaise, headache, fever, chills and muscle and joint pain, which are commonly reported in normal populations. These side effects often subside after a few days of injection. Acute thrombosis is a serious but very rare adverse event. It is related to any uncontrolled hypertension and blood glucose level. Amid concerns over possible cases of thrombosis after vaccination, the use of Astra Zeneca vaccine was stopped in several countries, and the order of this vaccine was suspended by the Hong Kong Government. In addition, currently available vaccines do not contain functional coronaviruses; thus, it is not possible to cause an active infection even in immunosuppressed patients.

In our study, cancer patients worried about the long-term side effects of the vaccine. As we all know, the COVID-19 vaccines are newly developed, and researchers are still learning any potential long-term side effects. Cancer patients have to understand the known high efficacy of vaccination and the unknown long-term side effects to make a sensible judgement in the current critical pandemic situation.

### 4.4. Focused Education on COVID-19 Vaccination Should Be Given to Cancer Patients and Their Care Providers

In our study, over 80% of the participants had education above the secondary level, and over 70% had a high cancer health literacy level (score of 5 or 6 in CHLT6). Surprisingly, they had a rather low level of vaccine knowledge, with over 80% of the participants getting none or 1–2 correct answers out of 5 questions in our quiz on vaccine knowledge. This shows that more public education on COVID-19 vaccination specific to cancer patients and their care providers should be given. Knowledge on the efficacy, tolerability and side effects of vaccines is wanted by the cancer patients and should be provided to both patients and carers. Our study also found that patients with better cancer health literacy were more likely to have a preference on the type of vaccine. Information about differences in vaccines (e.g., mRNA vaccine vs. inactivated whole virion vaccine) and mechanism should be given so that patients can make a good choice of vaccine suitable for their health.

We acknowledge certain limitations of this study. First, this is a cross-sectional study, which does not allow the assessment of the causality of different psychological outcomes. Second, only two institutions were involved in this study and convenience sampling was used. This might limit the generalizability of the results. Third, questionnaires were self-administered owing to strict infection control measures. Therefore, the information obtained was not verified by medical personnel. Fourth, the questionnaire may not be comprehensive and might not cover all concepts to explain the unwillingness of vaccination in cancer patients. For example, there is no assessment of the perception of cancer patients on existing preventive measures. A qualitative study with cancer patients may give more in-depth and detailed answers for the low acceptance rate among cancer patients. Fifth, self-reporting may be influenced by social desirability and may cause reporting bias. Sixth, the infection rate in cancer patients in Hong Kong is not known and these data might influence the vaccination rate. The associations between the infection rate and the acceptance of vaccination in cancer patients are not assessed in this study. Seventh, it would be interesting to repeat the study several months after the implementation of the vaccination programme. This would capture the up-to-date underlying reasons for the low vaccination rate among cancer patients in Hong Kong. However, this is out of the scope of this study.

## 5. Conclusions

We found a very low intended acceptance rate of COVID-19 vaccination in cancer patients in Hong Kong. Patients’ main concerns include the adverse effects of vaccines on cancer treatment as well as the short- and long-term side effects. Although larger studies over longer periods of follow-up are required to fully assess the efficacy and safety issues of COVID-19 vaccines, the decision to vaccinate patients who are vulnerable cannot be delayed while waiting for the long-term data from prospective clinical trials. International medical organizations and societies already published guidance on COVID-19 vaccination in cancer patients. Health educators and community health workers should provide knowledge about the vaccines and offer public education on how to protect from infection to improve vaccine uptake. Oncologists and health provision team should work together and offer individual counselling to cancer patients and care providers to relieve their anxiety. They need to change the focus of the cancer patients, turning them from worrying about the side effects of the vaccine and adverse impact of vaccine on cancer treatment, to acknowledging the need for protection from vaccination. More importantly, oncologists can help patients to make decisions on vaccination. COVID-19 vaccines are new to both the public and cancer patients. Educational sessions on COVID-19 with special focus on cancer patients’ concerns should be provided through different media platforms, such as the Internet, newspaper, radio and television.

## Figures and Tables

**Table 1:** Univariable analysis on clinical characteristics of participants on acceptance of COVID-19 vaccination.

Variables		Total	Participants Likely to Get a COVID-19 Vaccine		Odds Ratio	95% C.I.		*p*-Value
	*N* = 660		N = 118					
			17.9%					
	*n*	%	*n* (%)			Lower	Upper	
**Gender**								
Men	228	34.5%	54	(23.7)	ref			
Women	432	65.5%	64	(14.8)	0.56	0.37	0.84	**0.005**
**Age**								
18–60	358	54.2%	58	(16.2)	ref			
≥61	302	45.8%	60	(19.9)	1.28	0.861	1.911	0.221
**Marital status**								
Not married	190	28.8%	37	(19.5)	ref			
Married	470	71.2%	81	(17.2)	0.86	0.56	1.33	0.497
**Employment status**								
Not working	415	62.9%	73	(17.6)	ref			
Working	245	37.1%	45	(18.4)	1.05	0.699	1.59	0.801
**Education**								
Below tertiary level	412	62.4%	79	(19.2)	ref			
Tertiary level or above	248	37.6%	39	(15.7)	0.79	0.52	1.20	0.263
**Medical insurance**								
No	377	57.1%	78	(20.7)	ref			
Yes	283	42.9%	40	(14.1)	0.63	0.42	0.96	**0.030**
**Stage of disease**								
Stage I–III	276	41.8%	52	(18.8)	ref			
Stage IV	384	58.2%	66	(17.2)	0.90	0.60	1.34	0.585
**Currently on anticancer systemic treatment**								
No (Radiotherapy/survivors/palliative care)	204	30.9%	43	(21.1)	ref			
Yes (On anticancer systemic treatment)	456	69.1%	75	(16.4)	0.74	0.49	1.12	0.151
**Flu vaccination in the past 12 months**								
No	519	78.6%	91	(17.5)	ref			
Yes	141	21.4%	27	(19.1)	1.11	0.69	1.79	0.657
**CHLT-6 score**					0.79	0.68	0.91	**0.001**
**HADS Depression score**								
Normal/ borderline abnormal (score <11)	458	69.4%	94	(20.5)	ref			
Abnormal (score ≥11)	202	30.6%	24	(11.9)	0.52	0.32	0.85	**0.008**
**HADS Anxiety score**								
Normal/ borderline abnormal (score <11)	607	92.0%	108	(17.8)	ref			
Abnormal (score ≥11)	53	8.0%	10	(18.9)	1.08	0.52	2.21	0.845

C.I.: Confidence interval; CHLT-6: Cancer Health Literacy Test-6; HADS: Hospital Anxiety and Depression Score.

**Table 2:** Multivariable analysis on factors associated with acceptance of COVID-19 vaccination.

Variables	Total	Participants Likely to Get a COVID-19 Vaccine		Odds Ratio	95% C.I.		*p*-Value
	*N* = 660	N = 118					
		17.9%					
	*n*	*n* (%)			Lower	Upper	
**Gender**							
Men	228	54	(23.7)	ref			
Women	432	64	(14.8)	0.70	0.45	1.09	0.115
**Age**							
18–60	358	58	(16.2)	ref			
>61	302	60	(19.9)	0.93	0.59	1.47	0.746
**Medical insurance**							
No	377	78	(20.7)	ref			
Yes	283	40	(14.1)	0.65	0.41	1.04	0.071
**Stage of disease**							
Stage I–III	276	52	(18.8)	ref			
Stage IV	384	66	(17.2)	0.81	0.51	1.30	0.383
**Currently on anticancer systemic treatment**							
No (Radiotherapy/survivors/palliative care)	204	43	(21.1)	ref			
Yes (On anticancer systemic treatment)	456	75	(16.4)	0.62	0.38	1.01	0.056
Worry about getting COVID-19 infection							
No	147	28	(19.0)	ref			
Yes	513	90	(17.5)	1.12	0.65	1.92	0.692
**Worry more about cancer progression than COVID-19 infection**							
No	163	32	(19.6)	ref			
Yes	497	86	(17.3)	0.84	0.50	1.40	0.497
**Believe that COVID-19 vaccine can prevent COVID-19 infection**							
No	173	14	(8.1)	ref			
Yes	487	104	(21.4)	2.97	1.59	5.56	**0.001**
**Worry that COVID-19 vaccine will affect cancer treatment**							
No	184	38	(20.7)	ref			
Yes	476	80	(16.8)	1.09	0.65	1.84	0.733
**Worry about short-term side effects of vaccine**							
No	96	20	(20.8)	ref			
Yes	564	98	(17.4)	1.31	0.67	2.54	0.431
**Worry about long-term side effects of vaccine**							
No	192	53	(27.6)	ref			
Yes	468	65	(13.9)	0.46	0.28	0.76	**0.002**
**CHLT-6 score**				0.75	0.64	0.89	**0.001**
**HADS Depression score**							
Normal/borderline abnormal (score <11)	458	94	(20.5)	ref			
Abnormal (score ≥11)	202	24	(11.9)	0.49	0.29	0.82	**0.007**
**HADS Anxiety score**							
Normal/borderline abnormal (score <11)	607	108	(17.8)	ref			
Abnormal (score ≥11)	53	10	(18.9)	0.66	0.29	1.50	0.325

C.I.: Confidence interval; CHLT-6: Cancer Health Literacy Test-6; HADS: Hospital Anxiety and Depression Score.

**Table 3:** Multivariable analysis on factors associated with preference on type of vaccine (preference versus no preference).

Variables	Total	Participants Have Preference on Type of Vaccine		Odds Ratio	95% C.I.		*p*-Value
	*N* = 660	N = 376					
		57.0%					
	*n*	n (%)			Lower	Upper	
**Gender**							
Men	228	132	(57.9)	ref			
Women	432	244	(56.5)	0.68	0.47	0.97	**0.038**
**Age**							
18–60	358	219	(61.2)	ref			
>61	302	157	(52.0)	0.80	0.57	1.15	0.216
**Medical insurance**							
No	377	201	(55.3)	ref			
Yes	283	175	(61.8)	1.19	0.83	1.69	0.340
**Stage of disease**							
Stage I–III	276	145	(52.5)	ref			
Stage IV	384	231	(60.2)	1.23	0.86	1.75	0.261
**Currently on anticancer systemic treatment**							
No (Radiotherapy/survivors/palliative care)	204	133	(65.2)	ref			
Yes (On anticancer systemic treatment)	456	243	(53.3)	0.66	0.45	0.98	**0.038**
**Worry about getting COVID-19 infection**							
No	147	76	(51.7)	ref			
Yes	513	300	(58.5)	1.09	0.71	1.67	0.683
**Worry more about cancer progression than COVID-19 infection**							
No	163	96	(58.9)	ref			
Yes	497	280	(56.3)	0.98	0.66	1.48	0.938
**Believe that COVID-19 vaccine can prevent COVID-19 infection**							
No	173	92	(53.2)	ref			
Yes	487	284	(58.3)	1.16	0.79	1.70	0.454
**Worry that COVID-19 vaccine will affect cancer treatment**							
No	184	111	(60.3)	ref			
Yes	476	265	(55.7)	0.87	0.57	1.32	0.508
**Worry about short-term side effects of vaccine**							
No	96	51	(53.1)	ref			
Yes	564	325	(57.6)	1.39	0.81	2.39	0.235
**Worry about long-term side effects of vaccine**							
No	192	107	(55.7)	ref			
Yes	468	269	(57.5)	1.28	0.83	1.95	0.263
**CHLT-6 score**				1.23	1.07	1.41	**0.004**
**Knowledge about vaccine**				1.38	1.21	1.57	**0.001**
**HADS Depression score**							
Normal/borderline abnormal (score <11)	458	277	(60.5)	ref			
Abnormal (score ≥11)	202	99	(49.0)	0.64	0.44	0.92	**0.015**
**HADS Anxiety score**							
Normal/borderline abnormal (score <11)	607	346	(57.0)	ref			
Abnormal (score ≥11)	53	30	(56.6)	0.78	0.42	1.45	0.430

C.I.: Confidence interval; CHLT-6: Cancer Health Literacy Test-6; HADS: Hospital Anxiety and Depression Score.

## Data Availability

The data presented in this study are available on request from the corresponding author. The data are not publicly available due to patient’s privacy.

## Appendix A. Participant’s Questionnaire

**Introduction**

You are being invited to take part in a research study conducted by Department of Clinical Oncology of Queen Mary Hospital and the University of Hong Kong. Before you decide, it is important for you to understand the purpose and details of this research. Please feel free to ask if you need further details.

**Topic: Acceptance of COVID-19 vaccination in cancer patients in Hong Kong**

**Objective**

To understand the perception and conern of cancer patients on COVID-19 vaccination.

**Basic Information**

1. Gender: Male FMale 2. Age: 18–40 41–50 51–60 61–70 >70 3. Marriage: Single Married Divorced Widowed 4. Education Level: Primary or below Secondary Diploma Bachelor or above	5. Current Employment Status: Employed/Self-employed Retired Housewife Unemployed 6. Medical Insurance: Yes No

7. Type of Cancer (You can choose more than one answer.):
    Lung cancer
    Bowel cancer
    Gastric cancer, esophageal cancer, pancreatic cancer, liver cancer
    Breast cancer
    Head and neck cancer (including nasopharyngeal cancer)
    Skin cancer
    Thyroid cancer
    Gynecological cancer includes uterine cancer, cervical cancer, and ovarian cancer
    Urology cancer includes kidney cancer, bladder cancer, prostate cancer
    Hematological cancers include leukemia and lymphoma
    Sarcoma
    Others, please specify: ____________________
8. Stage of Disease:
    Stage I–III
    Stage IV
9. Currently receiving (You can choose more than one answer.):
    Recovered, no further treatment
    Anti-cancer systemic treatment (chemotherapy, hormonal therapy, targeted therapy, immunotherapy)
    Radiotherapy
    Palliative therapy
    Not yet received therapy
10. Did you receive flu vaccination in the past 12 months?
    Yes
    No
11 Have you ever felt uncomfortable after vaccination?
    Yes, please specify: ________________________
    No
    Not applicable

**Please answer the following questions, and tick (☑) the most suitable option.**

**(A) Views towards COVID-19 vaccines.**

	A Strongly disagree	B Disagree	C Agree	D Strongly agree
(1) I worry about getting COVID-19 infection.				
(2) I will have more severe disease compared to others if I get COVID-19 infection.				
(3) I need to stop anti-cancer treatment if I get COVID-19 infection.				
(4) My cancer management will be affected if I get COVID-19 infection.				
(5) I worry more about cancer progression than COVID-19 infection.				
(6) Vaccine can prevent me from COVID-19 infection.				
(7) Vaccine can reduce risk of COVID-19 complications.				
(8) I worry that vaccine will affect my anti-cancer treatment.				
(9) I worry about short-term side effects of vaccine.				
(10) I worry about long-term side effects of vaccine.				
(11) I wish to receive vaccination as soon as possible.				
(12) I wish to wait for some time after vaccine implementation.				
(13) Cancer patients should be prioritised to have vaccination.				

**(B) Basic Knowledge on COVID-19 Vaccination**

	A Sinovac	B BioNTech	C AstraZeneca	D Not sure
(1) Which of the following vaccines use “genetically modified messenger RNA (mRNA)” as a technology platform?				
(2) Which of the following vaccine uses a technical platform that previously known for hepatitis A vaccine?				
(3) Which of the following vaccines needs to be stored at extremely low temperature (−70 °C) to maintain effectiveness?				
(4) Which of the following vaccines has serious side effects including transverse myelitis?				
(5) Which of the following vaccines has a clinical protective effectiveness of 95%?				

To summarise, if free COVID-19 vaccination will be launched next month in Hong Kong, while you can choose one from three of the vaccines, i.e., Sinovac, BioNtech and AstraZeneca, how likely will you get vaccination?

  Very likely

  Not likely

  Fifty-fifty

  Quite likely

  Very likely

If you are planning to receive vaccination, and you can freely choose one from three of the COVID-19 vaccines available in Hong Kong, i.e., Sinovac, BioNtech and AstraZeneca, do you have preference on type of vaccine?

  I have preference

  I don’t have preference/ For me, those three types are the same

What information do you want to know before COVID-19 vaccination? (You can choose more than one answer.)

  Side effects of the vaccine

  Efficacy of the vaccine

  Effects of vaccine towards cancer treatment

**6-item Cancer Health Literacy Test (CHLT-6)**

**Please answer the following questions, and tick (☑) the most suitable option.**

1. The normal range for hemoglobin for a male is 13.3–17.2 g/dL. Joe’s hemoglobin is 9.7 g/dL. Is Joe within the normal range?
    Yes
    No
2. A biopsy of a tumor is done to:
    Remove it
    Diagnose it
    Treat it
3. If a patient has stage 1 cancer, it means the cancer is:
    Localized
    In nearby organs
    In distant sites
4. The role of a physical therapist is to talk to a patient about emotional needs.
    True
    False
5. A tumor is considered “inoperable” when it cannot be treated with:
    Radiation Therapy
    Surgery
    Chemotherapy
6. Sally will get radiation therapy once a day, Monday through Friday. If Sally has therapy for 4 weeks, how many times will she get radiation therapy?
    5
    15
    20

## Appendix B. Participant’s Characteristics

	Number	Percentage
Total number of patients	660	
Gender		
○ Male	228	34.5%
○ Female	432	65.5%
Age		
○ 18–40	51	7.7%
○ 41–50	130	19.7%
○ 51–60	177	26.8%
○ 61–70	188	28.5%
○ >70	114	17.3%
Marriage		
○ Not married	103	15.6%
○ Married	470	71.2%
○ Divorced	59	8.9%
○ Widowed	28	4.2%
Education		
○ Primary	104	15.8%
○ Secondary	308	46.7%
○ Tertiary	59	8.9%
○ University or above	189	28.6%
Employment status		
○ Employed	245	37.1%
○ Retired	237	35.9%
○ Housewife	120	18.2%
○ Unemployed	58	8.8%
Medical insurance		
○ Yes	283	42.9%
Type of cancer		
○ Lung	114	17.3%
○ Gastrointestinal	154	23.3%
○ Breast	192	29.1%
○ Head & neck	88	13.3%
○ Gynaecological	46	7.0%
○ Urological	30	4.5%
○ Sarcoma	7	1.1%
○ Other	29	4.4%
Stage of disease		
○ 1–3	276	41.8%
○ 4	384	58.2%
Currently on anticancer systemic treatment		
○ No (Radiotherapy/ survivors/ palliative care)	204	30.9%
○ Yes (On anticancer systemic treatment)	456	69.1%
Received flu vaccine in the past 12 months		
○ Yes	141	21.4%
Had discomfort after flu vaccine?		
○ Yes	41	6.2%
